# 

**Published:** 1902-08

**Authors:** 


					﻿LEADS VETERINARY JOURNALISM IN AMERICA.
Vol. XXIII.
AUGUST, 1902.
No. 8
PUBLISHED MONTHLY AT S3 A YEAR. SINGLE COPIES, 30 CENTS. .
FOREIGN SUBSCRIPTIONS $3-50 PER YEAR.
-«c
o_
LU
oc
a_
LU
cs
«<c
b—
C/3
a_
1
o
<
u
o
10
ci
6
2
o
o
0)
d
0)
1
co
0)
I
b-
0)
I
(0
0)
CD
LX.
C9
GO
a_
co
CJ
C3
CO
OQ
THE JOURNAL'
OF
Comparative Medicine
AND
VETERINARY ARCHIVES
EDITED AND PUBLISHED BY
W. HORACE HOSKINS, D.V.S.
ALL COMMUNICATIONS AND PAYMENTS SHOULD BE ADDRESSED TO
Dr. W. Horace Hoskins, Managing editor, 3452 Ludlow St;, Philadelphia, Pa.
Copies may be had on order of all news-dealers at home and abroad.
AN ADVERTISING MEDIUM UNEQUALLED IN THE VETERINARY FIELD
CO
CO
“O
-<
a
-T1
—I
m
o
c
©
z
I"
“n
O
©
(0
o
£
£
o
5
OT
CD
00
o
o
7
"H
SO
-4
•
o
o
				

